# Estimation of Genetic Parameters of Reproductive Traits in Holstein Cattle from Southern China

**DOI:** 10.3390/ani16040604

**Published:** 2026-02-14

**Authors:** Wenjie Li, Shuwen Xia, Yanming Quan, Yangyang Shen, Weining Li, Kunlin Chen, Zhenjiang An, Yingying Jiang, Zengxiang Pan, Huili Wang

**Affiliations:** 1 College of Animal Science and Technology, Nanjing Agricultural University, Nanjing 210095, China; 2023105025@stu.njau.edu.cn (W.L.);; 2 Jiangsu Provincial Engineering Research Center of Precision Animal Breeding, Institute of Animal Science, Jiangsu Academy of Agricultural Sciences, Nanjing 210014, Chinaliwn@jaas.ac.cn (W.L.);

**Keywords:** Holstein cow, reproductive traits, genetic parameter, heritability, southern China

## Abstract

Reproductive performance is a critical determinant of dairy farm productivity and profitability. This study aimed to estimate genetic parameters for key reproductive traits in Holstein cattle from southern China. Phenotypic data comprising 117,514 records from 44,861 cows across eight farms were analyzed. Genetic parameters were estimated separately for heifers and multiparous cows using single-trait animal and repeatability models via the AI-REML module in DMU. Bivariate and multi-trait models were employed to estimate trait correlations within and across parity groups, respectively. The results indicate that heifers generally exhibited superior reproductive efficiency compared to multiparous cows. Heritability estimates ranged from 0.04 to 0.47 in heifers, with age at first service (AFS) being the highest (0.47 ± 0.02), while estimates for multiparous cows were consistently low (0.03–0.14). Strong genetic correlations were observed among functionally related traits, particularly in multiparous cows. For example, high positive genetic correlations were detected between Number of Services (NSc) and Interval from First to Last Service (IFLc, 0.91), NSc and Days Open (DO, 0.87), Interval from First to Last Service (IFLc) and Calving Interval (CI, 0.94), and IFLc and DO (0.96). Multi-trait analysis revealed moderate genetic correlations (0.13–0.85) for the same trait across heifers and different cow parities, with gestation length (GL) showing the strongest correlation (0.99) across all parities. The findings confirm that most reproductive traits in this population, especially in multiparous cows, exhibit low heritability and are strongly influenced by management and environment. This study thereby delivers practical guidance for the genetic improvement of low-heritability traits and for the production management of Holstein cattle in southern China.

## Simple Summary

Reproductive performance directly affects the productivity and profit of dairy farms. To support region-specific breeding, this study estimated the genetic parameters for key reproductive traits in Holstein cattle from southern China. Analysis of over 117,000 records from 44,861 cows showed that heifers had better reproductive efficiency and higher genetic potential for these traits than multiparous cows. In multiparous cows, reproductive traits were found to have very low genetic heritability, meaning they are influenced more by farm management and environment than by genetics. This provides clear guidance: genetic selection for reproductive efficiency should be prioritized in heifer breeding programs, while for multiparous cows, investment should focus on optimizing nutritional, health, and environmental management to improve reproductive outcomes.

## 1. Introduction

Reproductive traits are critical functional characteristics in dairy cattle breeding [1], directly impacting calving intervals, lactation cycles, and herd renewal efficiency, thereby influencing farm productivity and profitability [2,3]. Among the key indicators used to assess reproductive development and efficiency in heifers are Age at First Service (AFS) and Age at First Calving (AFC), which reflect heifer maturation synchronization [4,5]. AFC is a defining moment that describes an important event in the cow’s life, as it marks the beginning of the dairy cow’s productive career [6,7]. Number of Services (NS) typically refers to the total instances of artificial insemination performed within a single estrous cycle or until conception is achieved. It serves as a key operational indicator for assessing both individual cow reproductive efficiency and overall herd reproductive management. NS is highly negatively correlated with Conception Rate at First Service (CR) and positively correlated with both Interval from First to Last Service (IFL) and Calving Interval (CI) [8,9,10]. Consequently, NS functions as a core variable that comprehensively reflects the efficiency of the entire reproductive management chain, from estrus detection to successful conception. These traits are influenced by both genetic and environmental factors [11], making genetic evaluation essential for developing effective breeding and management strategies [12].

The estimation of genetic parameters is fundamental to modern dairy cattle breeding [13,14]. Heritability and genetic correlations among traits provide key insights into the genetic architecture of reproduction and guide selection decisions [14,15,16]. Previous studies have primarily focused on estimating genetic parameters for Holstein cattle [17,18,19]. Schwarz et al. [20] reported heritability estimates ranging from 0.014 to 0.211 in German Holsteins, while corresponding values in Korean dairy cattle were between 0.10 and 0.17 [16]. In northern China, Guo et al. [21] estimated heritability ranges for reproductive traits in Beijing Holsteins to be between 0.034 and 0.100, and Chen et al. [22] reported heritability estimates ranging from 0.231 to 0.483 for Holstein heifers in Ningxia. However, studies on genetic parameters for Holstein populations in southern China remain relatively scarce [23]. China exhibits substantial north–south differences in climate, management practices, feed resources [24], and breeding history [25]. These environmental factors likely interact with genetic backgrounds [26], causing significant divergence in the phenotypic expression of reproductive traits [27]. Consequently, genetic parameters estimated from northern herds may not be directly applicable to southern populations.

Reproductive performance differs substantially between heifers and multiparous cows due to difference in physiological stage, disorder susceptibility, and genetic expression patterns [14,28]. Combining data from both groups may obscure trait-specific genetic patterns. Internationally, heifers and cows are commonly assessed separately in genetic evaluations to improve selection accuracy [10,14,28]. Physiologically, heifers are in a stage of ongoing growth and gradual maturation of reproductive function. Their growth capacity and ability to maintain pregnancy exhibit considerable genetic potential [22]. They generally outperform multiparous cows in reproductive metrics [14,29], warranting separate genetic analysis.

This study aims to estimate genetic parameters for key reproductive traits in southern Chinese Holstein cattle, with classified analyses for heifers and multiparous cows. Objectives included estimating variance components, heritability, and genetic correlations among traits, and evaluating genetic relationships across parities. This study can establish a genetic basis for tailored breeding strategies across regions and physiological stages, thereby facilitating the genetic improvement of low-heritability traits, and ultimately enhance the productivity and sustainability of dairy systems in southern China.

## 2. Materials and Methods

### 2.1. Phenotypic Data

Reproductive data from 2010 to 2024 were collected from eight large-scale dairy farms in southern China (Shanghai, Jiangsu, Zhejiang, Anhui, Fujian, and Yunnan). The study area encompasses a broad longitudinal gradient from the eastern coastal region to the southwestern inland region, covering longitudes from approximately 97° E to 123° E and latitudes from 21° N to 35° N. The dataset comprises 117,514 records from 44,861 Holstein cows, including insemination, pregnancy checks, and calving events, with 39,207 records from heifers and 78,307 records from multiparous cows. Cattle were housed in stall barns under an intensive confinement system. Cooling measures (fans and sprinklers) were implemented during summer. All animals were fed a total mixed ration (TMR) formulated according to the Chinese Feeding Standard for Dairy Cattle, based on corn silage, alfalfa hay, corn, soybean meal, and mineral supplements.

The reproductive traits analyzed in the study are shown in Table 1, along with their corresponding definitions. Given the distinct reproductive characteristics between heifers and multiparous cows, the reproductive data for these two groups were analyzed separately.

For the dichotomous trait CR, outcomes were coded as 0 (no confirmed pregnancy) or 1 (confirmed pregnancy). Records prior to the first calving were classified as parity 0 for heifers, while records for the first, second, and third calvings in multiparous cows were coded as parities 1, 2, and 3, respectively [30].

### 2.2. Quality Control of Reproductive Data

Data quality control was performed according to following criteria [31]: AFS: 270–900 days; NS/NSc: 1–10; IFL/IFLc: 0–260 days; GL: 240–330 days; CTFS: 19–210 days; AFC: 510–1080 days; CI: 280–610 days; DO: 19–340 days; BW/BWc: 24–70 kg. After quality control, the dataset was reduced from 117,514 records to 116,954 records.

### 2.3. Pedigree Information

Pedigree information was obtained for 80,794 Holstein cattle, comprising 2695 bulls and 78,099 cows. Among the 44,861 phenotyped individuals, 44,126 (98.4%) had parental records, and 43,982 (98.0%) had grandparental records. Overall, the pedigree completeness reached 85%. Offspring birth dates were cross-checked with parental birth dates, and unreasonable pedigree entries were either removed or corrected to meet the requirements of DMU [32] software (Version 6, release 5.6) for genetic evaluation. Missing parent or grandparent information was replaced with “0”.

### 2.4. Analysis of Fixed Effects

Reproductive traits in Holstein cattle are influenced by numerous non-genetic factors, including herd (H), birth year (BY), year of first service (FSY), birth season (BS), season of first service (FSS), calving season (CSE), parity (P), and inseminator (BRE). The classification levels for these non-genetic factors are shown in Table 2. In this study, factors potentially affecting the traits were first treated as fixed effects for analysis of variance, and only those found to be statistically significant were retained in the final genetic analysis model. The Generalized Linear Mixed Model (GLMM) in R (v 4.4.0) was employed to test the significance of fixed effects. The model is as follows:

$$Y_{ijklmnop}=H_{i}+BY_{j}+FSY_{k}+BS_{l}+FSS_{m}+CSE_{n}+P_{o}+BRE_{p}+\varepsilon_{ijklmnop}$$

where $Y_{ijklmnop}$ is the observed phenotypic value of the reproductive traits for the i-th herd, the j-th birth year, the k-th year of first service, the l-th birth season, the m-th season of first service, the n-th calving season, the o-th parity, and the p-th breeder. $H_{i}$ denotes the herd effect; $BY_{j}$ denotes the birth year effect; $FSY_{k}$ denotes the year of first service effect; $BS_{l}$ denotes the birth season effect; $FSS_{m}$ denotes the season of first service effect; $CSE_{n}$ denotes the calving season effect; $P_{o}$ denotes the parity effect; $BRE_{p}$ denotes the breeder effect; and $\varepsilon_{ijklmnop}$ represents the random residual error. A significance threshold of *p* < 0.05 was applied to determine which fixed effects should be included in the final model.

### 2.5. Genetic Evaluation Models

#### 2.5.1. Single-Trait Animal Model

A single-trait animal model was employed to estimate the genetic parameters for reproductive traits in heifers (AFS, AFC, IFL, NS, GL, BW, and CR). The model is defined as follows:

y=Xb+Za+e

where y is the vector of observed phenotypic values for the heifer traits; b is the vector of fixed effects; a is the vector of additive genetic effects; e is the vector of random residual effects; and X and Z are the corresponding design matrices for b and a, respectively.

#### 2.5.2. Single-Trait Repeatability Model

A single-trait repeatability model was used to estimate the genetic components for reproductive traits in multiparous cows (DO, CTFS, CI, IFLc, NSc, GLc, BWc, and CRc). This model is appropriate for traits with repeated measurements and is defined as:

$$y=X\beta +Za+Wp_{e}+e$$

where y is the vector of individual phenotypic values; X is the design matrix for fixed effects; β is the vector of fixed effects; a is the vector of additive genetic effects; Z is its corresponding design matrix; $p_{e}$ is the vector of permanent environmental effects; W is the corresponding design matrix; and e is the vector of residual effects.

#### 2.5.3. Bivariate Models

To estimate trait correlations separately for heifers and multiparous cows, bivariate models were applied in two sequential analyses.

Bivariate animal model for heifer traits:

$$\left[\begin{array}{c} y_{1}\\ y_{2} \end{array}\right]=\left[\begin{array}{cc} X_{1} & 0\\ 0 & X_{2} \end{array}\right]\left[\begin{array}{c} b_{1}\\ b_{2} \end{array}\right]+\left[\begin{array}{cc} Z_{1} & 0\\ 0 & Z_{2} \end{array}\right]\left[\begin{array}{c} a_{1}\\ a_{2} \end{array}\right]+\left[\begin{array}{c} e_{1}\\ e_{2} \end{array}\right]$$

Bivariate repeatability animal model for multiparous cow traits:

$$\left[\begin{array}{c} y_{1}\\ y_{2} \end{array}\right]=\left[\begin{array}{cc} X_{1} & 0\\ 0 & X_{2} \end{array}\right]\left[\begin{array}{c} b_{1}\\ b_{2} \end{array}\right]+\left[\begin{array}{cc} Z_{1} & 0\\ 0 & Z_{2} \end{array}\right]\left[\begin{array}{c} a_{1}\\ a_{2} \end{array}\right]+\left[\begin{array}{cc} W_{1} & 0\\ 0 & W_{2} \end{array}\right]\left[\begin{array}{c} p_{e1}\\ p_{e2} \end{array}\right]+\left[\begin{array}{c} e_{1}\\ e_{2} \end{array}\right]$$

In these expressions, $y_{i}$ represents the vector of phenotypic values for the i-th trait; $b_{i}$ is the vector of fixed effects for the i-th trait; $a_{i}$ is the vector of additive genetic effects for the i-th trait; $p_{e}$ is the vector of permanent environmental effects for the i-th trait; $e_{i}$ is the vector of random residual effects for the i-th trait; and $X_{i}$, $Z_{i},$ and $W_{i}$ are the corresponding design matrices.

#### 2.5.4. Multi-Trait Models

To analyze reproductive traits across multiple parities, multi-trait models were employed, in which the same trait recorded in different parities was treated as multiple distinct traits.

Four-trait model for traits with records in both heifers and multiparous cows, including NS, CR, IFL, GL and BW:

$$\left[\begin{array}{c} y_{0}\\ y_{1}\\ y_{2}\\ y_{3} \end{array}\right]=\left[\begin{array}{cccc} X_{0} & 0 & 0 & 0\\ 0 & X_{1} & 0 & 0\\ 0 & 0 & X_{2} & 0\\ 0 & 0 & 0 & X_{3} \end{array}\right]\left[\begin{array}{c} \beta 0\\ \beta 1\\ \beta 2\\ \beta 3 \end{array}\right]+\left[\begin{array}{cccc} Z_{0} & 0 & 0 & 0\\ 0 & Z_{1} & 0 & 0\\ 0 & 0 & Z_{2} & 0\\ 0 & 0 & 0 & Z_{3} \end{array}\right]\left[\begin{array}{c} a_{0}\\ a_{1}\\ a_{2}\\ a_{3} \end{array}\right]+\left[\begin{array}{c} e_{0}\\ e_{1}\\ e_{2}\\ e_{3} \end{array}\right]$$

Here, subscript 0, 1, 2, and 3 represent the parity groups (0 for heifers, and 1, 2, and 3 for first, second, and third parity in multiparous cows, respectively).

Three-trait model for traits with records only in multiparous cows including CTFS, CI, and DO:

$$\left[\begin{array}{c} y_{1}\\ y_{2}\\ y_{3} \end{array}\right]=\left[\begin{array}{ccc} X_{1} & 0 & 0\\ 0 & X_{2} & 0\\ 0 & 0 & X_{3} \end{array}\right]\left[\begin{array}{c} \beta 1\\ \beta 2\\ \beta 3 \end{array}\right]+\left[\begin{array}{ccc} Z_{1} & 0 & 0\\ 0 & Z_{2} & 0\\ 0 & 0 & Z_{3} \end{array}\right]\left[\begin{array}{c} a_{1}\\ a_{2}\\ a_{3} \end{array}\right]+\left[\begin{array}{c} e_{1}\\ e_{2}\\ e_{3} \end{array}\right]$$

In this model, subscript 1, 2, and 3 represent the first, second, and third parity in multiparous cows.

In the expressions above, $y_{i}$ represents the phenotypic value vector for the i-th trait; $a_{i}$ is the vector of additive genetic effects for the i-th trait; $e_{i}$ is the vector of random residual effects for the i-th trait; and $X_{i}$ and $Z_{i}$ are the corresponding design matrices.

### 2.6. Estimation of Genetic Parameter

Based on the variance and covariance components estimated by the DMU software (Version 6, release 5.6) [32], the heritability ($h^{2}$), repeatability ($r_{e}$), genetic correlation ($r_{a}$), and phenotypic correlation ($r_{p}$) for each trait were calculated with the following formulas:

Heritability:

$$h^{2}=\frac{\sigma_{a}^{2}}{\sigma_{p}^{2}}$$

where $\sigma_{a}^{2}$ is the additive genetic variance for the trait, and $\sigma_{p}^{2}$ is the phenotypic variance. For heifers: $\sigma_{p}^{2}=\sigma_{a}^{2}+\sigma_{e}^{2}$. For multiparous cows: $\sigma_{p}^{2}=\sigma_{a}^{2}+\sigma_{pe}^{2}+\sigma_{e}^{2}$. Here, $\sigma_{e}^{2}$ is the residual variance, and $\sigma_{pe}^{2}$ is the permanent environmental variance.

Repeatability:

$$r_{e}=\frac{\sigma_{a}^{2}+\sigma_{pe}^{2}}{\sigma_{a}^{2}+\sigma_{pe}^{2}+\sigma_{e}^{2}}$$

Genetic correlation:

$$r_{a}=\frac{\mathrm{cov}(a_{1},a_{2})}{\sqrt{\sigma_{a1}^{2}\cdot \sigma_{a2}^{2}}}$$

where $\mathrm{cov}(a_{1},a_{2})$ is the additive genetic covariance between trait 1 and trait 2; $\sigma_{a1}^{2}$ and $\sigma_{a2}^{2}$ are the additive genetic variances of trait 1 and trait 2, respectively.

Phenotypic correlation:

$$r_{p}=\frac{\mathrm{cov}(p_{1},p_{2})}{\sqrt{\sigma_{p1}^{2}\cdot \sigma_{p2}^{2}}}$$

where $\mathrm{cov}(p_{1},p_{2})$ is the phenotypic covariance between trait 1 and trait 2; $\sigma_{p1}^{2}$ and $\sigma_{p2}^{2}$ are the phenotypic variances of trait 1 and trait 2, respectively.

## 3. Results

### 3.1. Descriptive Statistics of Reproductive Traits

**Figure 1:**
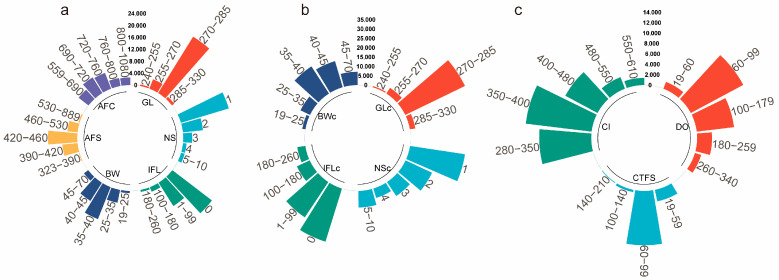
Statistical analysis of phenotypic data in Holstein cattle. (**a**) Hierarchical distribution of phenotypic data for six reproductive traits in heifers; (**b**,**c**) hierarchical distribution of phenotypic data for seven reproductive traits in multiparous cows.

Phenotypic data for heifers and multiparous cows for most reproductive traits followed a normal distribution, excluding the NS/NSc and IFL/IFLc traits (Figure 1). Summary statistics are presented in Table 3. Heifers showed relatively small variability, as indicated by their low coefficients of variation (CV). GL showed the smallest CV among all traits, while NS was primarily 1–2 services (91.5%), and IFL was mainly 0–99 d (89.7%).

In contrast, multiparous cows exhibited greater phenotypic variability for several traits, as evidenced by higher CV values. Although GLc remained within 270–285 d, NSc frequently extended to a third service (18.7% of records vs. 4.2% in heifers). BWc shifted toward 35–45 kg (78.9% of records), with a higher proportion exceeding 45 kg (11.3% vs. 3.6% in heifers). DO ranged from 60 to 179 d (76.4%), CTFS was 60–99 d, and CI spanned 280–480 d (89.2%).

**Figure 2:**
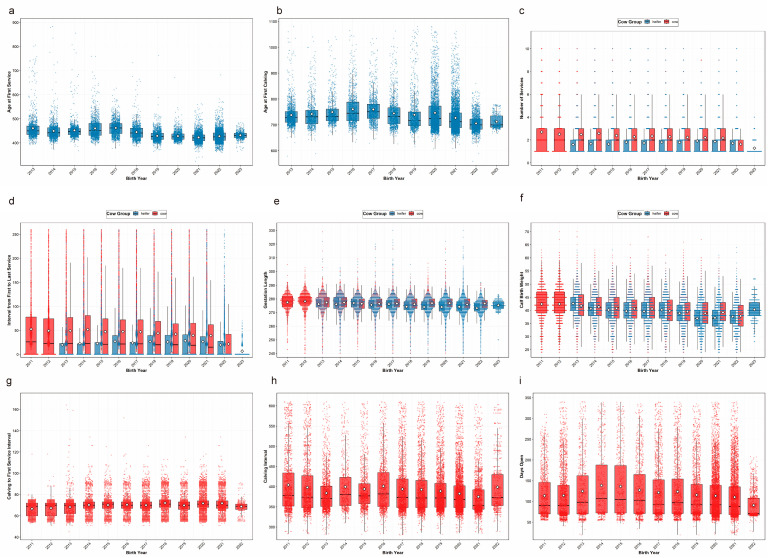
Distribution of reproductive phenotypes in heifers and multiparous cows over years of birth. (**a**) AFS; (**b**) AFC; (**c**) NS/NSc; (**d**) IFL/IFLc; (**e**) GL/GLc; (**f**) BW/BWc; (**g**) CTFS; (**h**) CI; (**i**) DO.

Figure 2 illustrates the temporal distribution of key reproductive phenotypes in heifers (born 2013–2023) and multiparous cows (born 2011–2022) across southern China. Phenotypic distributions differ markedly between heifers and multiparous cows in key reproductive traits (Figure 2c–f).

In heifers, AFS, AFC and IFL showed a steady downward trend over the study period. GL stayed stable within the 270–280 d range throughout the period, and BW stabilized at a moderate 35–40 kg from 2018 onward, reflecting consistent reproductive performance optimization. For multiparous cows, notable positive shifts were evident: NSc, IFLc, CI and DO exhibited a downward trend. Similar to heifers, GLc maintained stability at 270–280 d. CTFS remained consistent at around 70 d, and BWc stabilized near 38 kg after 2013.

### 3.2. Genetic Parameters and Variance Components of Reproductive Traits

Heritability estimates for reproductive traits in heifers ranged from 0.04 to 0.47 (Table 4). AFS showed the highest heritability (0.47 ± 0.02), followed by GL (0.19 ± 0.03) and BW (0.19 ± 0.03). NS (0.14 ± 0.01) and AFC (0.12 ± 0.02) displayed moderate heritability. CR (0.08 ± 0.01) and IFL (0.04 ± 0.01) were low-heritability traits. Based on these estimates, AFS was classified as a high-heritability trait (*h*^2^ > 0.30), whereas NS, GL, AFC, and BW were of medium heritability (0.10 < *h*^2^ ≤ 0.30). CR and IFL were considered low-heritability traits (*h*^2^ ≤ 0.10).

For multiparous cows, heritability estimates ranged from 0.03 to 0.14, with repeatability coefficients between 0.05 and 0.36 (Table 5). GLc (0.14 ± 0.03) and BWc (0.12 ± 0.03) exhibited medium heritability, whereas CRc, NSc, IFLc, CTFS, CI, and DO were low-heritability traits (*h*^2^ ≤ 0.10). Repeatability was highest for CRc (0.36) and lowest for CTFS and DO (both 0.05).

### 3.3. Genetic and Phenotypic Correlations of Reproductive Traits

**Figure 3:**
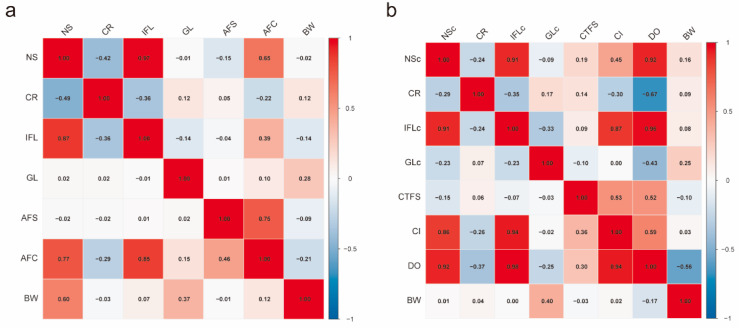
Genetic and phenotypic correlations among traits in Holstein cattle. (**a**) Genetic and phenotypic correlations among traits in heifers based on a bivariate model; (**b**) genetic and phenotypic correlations among traits in multiparous cows based on a bivariate repeatability model. Note: The upper triangle represents genetic correlations; the lower triangle represents phenotypic correlations.

Genetic and phenotypic correlations among reproductive traits in heifers and multiparous cows are summarized in Figure 3. In heifers, genetic correlations ranged from −0.42 to 0.97, and phenotypic correlations from −0.49 to 0.87 (Figure 3a). Notably, high genetic correlations existed between NS and IFL, NS and AFC, as well as between AFC and AFS, with absolute values ranging from 0.65 to 0.97. Phenotypically, NS was strongly correlated with IFL, AFC, and calf birth weight (BW), with absolute values reaching 0.87. CR exhibited a moderate negative phenotypic correlation with NS (−0.49).

For multiparous cows, genetic correlations ranged from −0.67 to 0.96 (Figure 3b). High positive genetic correlations were observed between NSc and IFLc, NSc and DO, IFLc and CI, and IFLc and DO, with values between 0.87 and 0.96. Phenotypic correlations spanned −0.37 to 0.98, with IFLc showing particularly high phenotypic associations with NSc (0.91), CI (0.94), and DO (0.98).

Regarding phenotypic correlations, the range was −0.37 to 0.98. IFLc showed relatively high phenotypic correlations with NSc, CI, and DO, ranging from 0.91 to 0.98.

### 3.4. Correlation Coefficients Between Different Parity Levels for the Same Trait Based on a Multi-Trait Model

Genetic and phenotypic correlations between different parity levels for the same trait were estimated using a multi-trait model and are presented in Table 6. Phenotypic correlations were generally low (0.00–0.40) between heifers and multiparous cows, as well as among parities within multiparous cows.

Genetic correlations were strongly positive for GL between heifers and all three parities in multiparous cows (0.99) and among parities within multiparous cows (0.99). For other reproductive traits, genetic correlations between heifers and multiparous cows were moderate, ranging from 0.13 (between heifer IFL and multiparous cows at third parity) to 0.85 (between heifer GL and multiparous cows GLc across all parities). However, higher genetic correlations were observed among different parity levels within multiparous cows, ranging from 0.40 to 0.99 for most traits, except for CTFS, CI, and DO, where genetic correlations between the first parity and the other two parities were below 0.40. Notably, genetic correlations between heifers and first-parity cows were generally higher than those between heifers and later-parity cows. Negative phenotypic correlations were observed for traits such as NS, IFL, and DO across different parity levels.

## 4. Discussion

### 4.1. Overview of Reproductive Performance in Holstein Cattle from Southern China

This study investigated reproductive traits of Holstein cattle across multiple provinces in southern China. Heifers generally exhibited superior reproductive efficiency compared to multiparous cows, as reflected by a shorter IFL, lower NS, and lower BW. These findings align with previous studies [8,33,34], indicating better conception potential in heifers. Since heifers have not yet undergone lactational stress or postpartum metabolic challenges, their reproductive systems remain in an optimal physiological state. The observed differences between heifers and multiparous cows may be attributed to physiological changes in the reproductive system after first calving and the negative energy balance associated with high milk production [17,30,35].

Heifers exhibited earlier AFS and AFC in this study compared to previous studies in Beijing [21], Ningxia [22] and the southern part of China [23]. This may be attributed to earlier sexual maturity in southern China, as well as management strategies on farms that emphasize early breeding and calving in heifer rearing [36,37]. For multiparous cows, the DO (118 days) was consistent with reports from Beijing [21] and Shandong [13] in China, and from Denmark [38], but shorter than previous findings in southern China [23] (approximately 157 days). Given that heat stress has been shown to prolong DO [39], this improvement likely reflects enhanced farm management (e.g., cooling facilities and nutritional adjustments) mitigating summer heat stress in the region [40,41].

The mean CI was 392.10 ± 63.84 d, which is similar to previous studies [42,43], such as those from Beijing (397.60 ± 60.0 d) [21] and Canada (395.4–398.2 d), but shorter than reported for Jiangsu (421.14 ± 80.07 d) [44] and an earlier study in southern China (429.19 ± 100.05 d) [23], indicating optimized postpartum reproductive recovery. In this study, the mean GL was 274 d for heifers and 276 d for multiparous cows, consistent with global and domestic benchmarks for Holstein cattle [14,17,23].

### 4.2. Heritability of Reproductive Traits

In this study, the estimated heritability for reproductive traits in Holstein cattle from southern China ranged from low to moderate. Except for AFS (0.47) in heifers, the heritability for other reproductive traits is at a low-to-moderate level (0.03–0.19), which is consistent with findings from other studies [33,45].

Heifer AFS exhibited a notably high heritability (0.47), exceeding estimates from Canadian (0.12–0.13) [46], German [47], and other southern Chinese Holstein populations (0.06) [23]. This discrepancy may be attributed to the strictly controlled rearing environment for heifers on the studied farms, where standardized nutrition, disease management, and housing conditions likely reduce the environmental variance component for AFS [48]. Additionally, differences in statistical methods and management strategies may also contribute to variations in heritability estimates [49,50].

In contrast, the heritability of traits in multiparous cows is generally lower than those for heifers, consistent with the previous studies [46,51]. For instance, heritability for NS (0.14) and CR (0.08) was higher in heifers than in multiparous cows (NSc: 0.03; CRc: 0.07), which aligns with findings in German [52] and northern Chinese studies [51]. Our estimates for CR/CRc (0.08 and 0.07) were moderately higher than those from Nordic [14] and Dutch populations [53]. These variations may arise from differences in genetic variation between populations, the statistical models employed for analysis, or genotype–environment interactions [54,55]. Notably, GL and BW both exhibit moderate heritability (0.14–0.19) in both heifers and multiparous cows, suggesting appreciable genetic influence and potential for genetic improvement.

For traits exclusive to multiparous cows, the heritability for CTFS and DO was 0.08 and 0.05, respectively. Similar results have been reported for Iranian [56,57] and South African Holsteins [29], but our results are higher than estimates from Tunisian [58] and northern Chinese Holsteins [21]. The heritability for DO (0.05) and CI (0.07) was close but not identical, whereas Iranian results reported very similar values of 0.069 and 0.070 for DO and CI, respectively [57].

These results reveal significant differences in the genetic background of reproductive traits between heifers and multiparous cows, supporting the need for group-specific breeding strategies. Heifers possess greater genetic potential for reproductive improvement; selection for moderate heritability in heifers, such as AFS, AFC, NS, GL, and BW, could enhance early reproductive efficiency. Physiologically, heifers, having not yet undergone lactational stress or postpartum metabolic disorders, maintain an optimal reproductive state [35]. In contrast, most reproductive traits in multiparous cows generally exhibit low heritability and are highly susceptible to environmental influences [19,29,38]. Differences in rumen microbiome [59,60], metabolism [35,40], and management [39,41] across parities may explain the heritability differences between heifers and multiparous cows. For example, heifers exhibit a more stable rumen microbial structure [61], whereas multiparous cows undergo microbiota disruption due to lactation and metabolic shifts [60], indirectly impairing reproductive efficiency [59]. However, direct evidence linking rumen microbiome composition to reproductive trait variation across parities in Holstein cows in southern China remains limited, necessitating further research to confirm this mechanism.

### 4.3. Genetic and Phenotypic Correlations of Reproductive Traits

High genetic correlations among different reproductive traits reveal a similar genetic background, enabling indirect selection for correlated traits [29]. In this study, a high genetic correlation (0.75 ± 0.05) was observed between AFC and AFS in heifers. This result aligns with findings from Jagusiak and Zarnecki [62] and Brzáková et al. [63]. As both AFS and AFC are traits specific to heifers and collectively reflect their reproductive performance level [23], they may share a common physiological basis. Furthermore, the high positive genetic correlation between IFL and NS is likely due to the delayed timing of the final service caused by an increased number of inseminations, which is also reflected in the high phenotypic correlation between these two traits.

For multiparous cows, DO exhibited high genetic correlations (0.45–0.96) with several traits, including NSc, IFLc, and CI. The highest genetic correlation was observed between DO and IFLc (0.96), suggesting that DO could potentially substitute for IFLc in genetic evaluations when farm insemination records are incomplete [13,21]. Furthermore, high genetic correlations were found among traits specific to multiparous cows, reflecting their ability to conceive, maintain pregnancy, and return to estrus postpartum. The genetic correlations were 0.53 between CTFS and CI, 0.52 between CTFS and DO, and 0.59 between CI and DO. These estimates align with reports in Chinese [21,30] and New Zealand [64] Holstein populations. Phenotypic correlations among CTFS, DO, and CI were also strong (0.30–0.94), supporting the close biological linkage among these intervals, which aligns with previous studies [21,29,65]. In practice, using CTFS as a proxy for DO can minimize data errors, as DO excludes cows culled for infertility, and CTFS is available earlier. Shorter CTFS and DO offer a shorter CI, which can prolong the lactation period, thereby improving productivity and herd profitability [66].

Biologically phenotypic correlations were observed, and their patterns were like those of the genetic correlations for all traits. Very strong positive relationships of 0.98 and 0.94 were identified between DO and IFLc, and between DO and CI, respectively, meaning that when one of these traits decreases, the other tends to decrease as well. A negative phenotypic relationship (−0.37) was found between CR and DO, which is similar to the estimate found in Korean dairy cattle (−0.66) [8] and Chinese dairy cattle (−0.75) [13], reflecting the expected trade-off between reproductive and postpartum anestrus.

### 4.4. Genetic Relationships Across Parities Provide a Basis for Early Selection

In general, moderate genetic correlations were observed between heifer traits and their corresponding traits in multiparous cows, ranging from 0.13 (between heifer IFL and third-parity IFLc) to 0.85 (between heifer GL and GLc in the first, second, and third parity). This finding is consistent with reports by Pryce et al. [42], Norman et al. [45], and Gredler and Schnyder [67].

Although heifer reproductive traits are phenotypically distinct from those of multiparous cows, they are genetically correlated to a certain extent, a pattern also noted in Nordic Holsteins and red cattle [14]. For most traits, correlations among parities 1–3 ranged from 0.40 to 0.99, except for CTFS, CI, and DO, which showed lower correlations (<0.40) between first and later parity. Notably, genetic correlations were typically higher between heifers and first-parity cows than between heifers and higher-parity cows.

CR/CRc exhibited genetic correlations of 0.50 to 0.91 across parities, which is similar to Liu et al.’s estimate of 0.6 between specific parities [51] but lower than Nordic estimate of 0.90–0.97 [14], reflecting population or modeling differences. Meanwhile, gestation length (GL/GLc) showed near-unity genetic correlations (up to 0.99) across all parities, indicating it can be treated as the same trait genetically regardless of parity. In contrast, genetic correlation between NS and CR was low (0.03–0.47) between heifers and multiparous cows, indicating that these traits may be regulated by parity-specific genes and should be treated differently in genetic evaluation. These findings hold significant implications for genomic selection and early breeding: reproductive traits in heifers can serve as early indicators for predicting future reproductive performance [47]. Particularly for traits with high genetic correlations, such as GL and BW, where early selection in heifers can effectively enhance lifetime reproductive efficiency. Conversely, traits with low cross-parity correlations may require parity-specific breeding objectives or evaluation models.

### 4.5. Influence of Environment and Management on Reproductive Traits and Breeding Implications

The hot and humid climate of southern China constitutes a major environmental challenge to dairy cow reproductive performance. The low heritability estimates for most reproductive traits in multiparous cows observed highlight the substantial contribution of environmental variance, with heat stress being a particularly key factor [68,69]. Heat stress can extend DO and CI through multiple pathways, including suppression of estrus expression, impairment of oocyte competence [70,71], and compromised embryo survival [72]. Consistent with this, previous studies indicate that each unit increase in the temperature–humidity index can prolong Days Open by 5–10 days [39]. Therefore, genetic selection for improved reproductive in southern China should be integrated with active environmental management strategies.

To bridge the research outcomes with practical breeding applications, a differentiated selection strategy is proposed for heifers and multiparous cows. For heifer breeding programs, prioritize genetic selection for traits with moderate-to-high heritability (e.g., AFS) by incorporating estimated breeding values (EBVs) into selection indices. In contrast, reproductive traits in multiparous cows typically demonstrate low heritability (<0.10), leading to limited annual genetic gain. However, substantial genetic variance in multiparous cows indicates considerable genetic potential remains. In practice, for multiparous cows, management interventions, including optimized formulas and environment control, should be prioritized to minimize the impact of non-genetic factors on their reproductive performance. Practical measures such as implementing cooling systems [73,74] (e.g., spray-and-fan ventilation), scheduling breeding to avoid peak heat periods [69], and formulating diets that enhance thermoregulation and mitigate heat stress are essential to support genetic potential and optimize reproductive outcomes.

## 5. Conclusions

This study presents a comprehensive genetic analysis of key reproductive traits in Holstein cattle from southern China, with separate evaluations for heifers and multiparous cows. The main findings demonstrate that reproductive performance in heifers is generally superior to that in multiparous cows, as evidenced by shorter breeding intervals and fewer services required. Genetic parameter estimates revealed low-to-moderate heritability for most traits, indicating substantial environmental influence. Notably, significant genetic differences were observed between heifers and multiparous cows for the same traits, supporting their treatment as distinct traits in genetic evaluations. Strong genetic correlations were identified among functionally related traits, particularly between CI and DO in multiparous cows. These findings have important implications for breeding and management strategies in southern China. Given the low heritability of reproductive traits, genetic improvement should emphasize multi-trait selection indices that incorporate reproductive traits with other economically important characteristics. Simultaneously, management practices, particularly those addressing heat stress mitigation in the region’s challenging climate, are essential to optimize reproductive performance and enhance overall herd productivity.

## Figures and Tables

**Table 1:** Reproductive traits analyzed separately for heifers and multiparous cows.

Trait Abbreviation	Full Name	Definition (Units)	Analyzed in
AFS	Age at First Service	Interval between birth date and first service date (days)	Heifers
AFC	Age at First Calving	Interval between birth date and first calving date (days)	Heifers
CR/CRc	Conception Rate at First Service	Proportion of successful pregnancies from first service (0/1)	Heifers/multiparous cows
NS/NSc	Number of Services	Total service attempts from first to successful service (count)	Heifers/multiparous cows
IFL/IFLc	Interval from First to Last Service	Period between first and successful service within parity (days)	Heifers/multiparous cows
GL/GLc	Gestation Length	Duration from successful service to subsequent calving (days)	Heifers/multiparous cows
BW/BWc	Calf Birth Weight	Newborn calf weight measured after birth (kg)	Heifers/multiparous cows
CTFS	Calving to First Service Interval	Period from calving to first subsequent service (days)	Multiparous cows
CI	Calving Interval	Interval between two consecutive calving dates (days)	Multiparous cows
DO	Days Open	Interval from calving to a successful subsequent service (days)	Multiparous cows

Note: The suffix “c” denotes the corresponding traits in multiparous cows, distinguishing them from those in heifers.

**Table 2:** Non-genetic factors and their levels for reproductive traits in Holstein cows.

Non-Genetic Factors	Level Classification	
	Heifers	Multiparous Cows
Herd (H)	8 levels (1 level per pasture)	8 levels (same as heifers)
Birth Year (BY)	2013–2023 (11 levels, 1 level per year)	2011–2022 (12 levels, 1 level per year)
First Service Year (FSY)	2014–2024 (11 levels, 1 level per year)	2012–2024 (13 levels, 1 level per year)
Birth Season (BS)	4 levels: spring (March–May), summer (June–August), fall (September–November), winter (December–February)	4 levels (same as heifers)
First Service Season (FSS)	4 levels (same classification criteria as birth season)	4 levels (same as heifers)
Calving Season (CSE)	4 levels (same as birth season classification criteria)	4 levels (same as heifers)
Parity (P)	---	7 levels: 1–6 parities (1 level per parity), more than 6 parities (1 level)
Breeder (BRE)	75 levels (1 level per breeder)	81 levels (varies by breeder count)

**Table 3:** Descriptive statistics of reproductive traits in heifers and multiparous cows.

Traits	N	Max	Min	Mean	SD	CV%
AFS	18,953	889	323	439.00	36.96	8.40
AFC	21,237	1080	559	735.80	62.43	8.48
CR/CRc	33,558/66,933	1	0	0.64/0.68	0.08/0.07	12.50/10.29
NS/NSc	28,953/68,119	10	1	1.79/2.40	0.28/0.83	15.64/34.58
IFL/IFLc	28,197/64,089	260	0	26.20/48.18	6.67/14.62	25.46/30.34
GL/GLc	26,943/42,469	330	240	274.80/276.50	6.79/6.80	2.47/2.46
BW/BWc	25,537/46,021	70	24	37.85/39.25	5.76/6.91	15.22/17.61
CTFS	13,221	207	19	71.51	14.78	20.67
CI	33,498	610	280	392.10	33.84	8.63
DO	24,128	340	19	118.50	24.71	20.85

**Table 4:** Variance components and genetic parameters of reproductive traits in heifers.

	VC	*σ* ^2^ *_a_*	*σ* ^2^ *_e_*	*h* ^2^	SE
Traits					
AFS		345.84	389.15	0.47	0.02
CR		0.01	0.11	0.08	0.01
NS		0.22	1.32	0.14	0.01
IFL		77.19	1733.67	0.04	0.01
GL		9.16	38.82	0.19	0.03
AFC		344.77	2565.70	0.12	0.02
BW		9.16	38.82	0.19	0.03

**Table 5:** Variance components and genetic parameters of reproductive traits in multiparous cows.

	VC	*σ* ^2^ *_a_*	*σ* ^2^ *_pe_*	*σ* ^2^ *_e_*	*h* ^2^	SE	*r_e_*
Traits							
CRc		0.01	0.04	0.09	0.07	0.02	0.36
NSc		0.05	0.09	1.38	0.03	0.01	0.09
IFLc		44.74	176.36	1387.42	0.03	0.02	0.14
GLc		5.66	1.53	33.51	0.14	0.03	0.18
CTFS		5.82	0.00	70.51	0.08	0.02	0.05
CI		51.52	0.00	652.78	0.07	0.02	0.07
DO		29.04	0.00	597.61	0.05	0.02	0.05
BWc		6.13	10.34	32.77	0.12	0.03	0.33

**Table 6:** Correlation coefficients of the same trait across different parities based on the multi-trait model.

Traits	NS	CR	IFL	GL	BW	CTFS	CI	DO
N	33,840	423,250	40,866	24,956	25,690	15,516	11,899	15,596
*r_a_*_0*,a*1_	0.07 (0.12)	0.47 (0.11)	0.34 (0.17)	0.99 (0.20)	0.73 (0.08)	-	-	-
*r_a_*_0*,a*2_	0.24 (0.17)	0.03 (0.16)	0.15 (0.38)	0.99 (1.34)	0.85 (0.09)	-	-	-
*r_a_*_0*,a*3_	0.06 (0.22)	0.08 (0.22)	0.13 (0.34)	0.99 (1.39)	0.66 (0.22)	-	-	-
*r_a_*_1*,a*2_	0.61 (0.17)	0.50 (0.13)	0.82 (0.35)	0.99 (1.70)	0.81 (0.09)	−0.03 (0.12)	0.08 (0.29)	−0.20 (0.41)
*r_a_*_1*,a*3_	0.89 (0.20)	0.63 (0.19)	0.56 (0.29)	0.99 (1.46)	0.96 (0.18)	0.25 (0.70)	0.35 (0.30)	0.04 (0.80)
*r_a_*_2*,a*3_	0.89 (0.26)	0.91 (0.22)	0.82 (0.57)	0.99 (2.81)	0.81 (0.21)	0.40 (0.89)	0.81 (0.53)	0.97 (1.16)
*r_p_*_0*,p*1_	−0.06 (0.09)	0.02 (0.22)	−0.11 (0.31)	0.12 (0.07)	0.14 (0.08)	-	-	-
*r_p_*_0*,p*2_	−0.03 (0.12)	0.15 (0.06)	−0.07 (0.04)	0.12 (0.13)	0.12 (0.16)	-	-	-
*r_p_*_0*,p*3_	−0.08 (0.11)	0.17 (0.13)	−0.08 (0.04)	0.09 (0.12)	0.12 (0.04)	-	-	-
*r_p_*_1*,p*2_	−0.08 (0.11)	0.17 (0.13)	−0.04 (0.12)	0.17 (0.07)	0.36 (0.21)	−0.09 (0.31)	−0.14 (0.04)	−0.16 (0.12)
*r_p_*_1*,p*3_	0.06 (0.14)	0.28 (0.13)	−0.04 (0.09)	0.14 (0.18)	0.33 (0.06)	0.26 (0.12)	0.10 (0.03)	−0.09 (0.35)
*r_p_*_2*,p*3_	0.07 (0.22)	0.23 (0.21)	0.01 (0.12)	0.19 (0.22)	0.40 (0.21)	0.33 (0.21)	0.16 (0.14)	−0.30 (0.28)

Note: $r_{a_{j},a_{i}}$ = genetic correlation between parity i and parity j for the same trait; $r_{p_{j},p_{i}}$ = phenotypic correlation between parity i and parity j for the same trait. Values in parentheses are standard errors for correlations. For traits NS, CR, IFL, GL, and BW, the sample size represents the total across parities 0 (heifers), 1, 2, and 3. For traits CTFS, CI, and DO, the sample size represents the total across parities 1, 2, and 3.

## Data Availability

The datasets generated and analyzed during the current study are available from the corresponding author on reasonable request.
